# Early Osseointegration in a Sheep Tibia Model: Correlating Digital Periapical Radiograph Gray-Level and RGB-Derived Metrics with Histologic Tissue Composition

**DOI:** 10.3390/jfb16110415

**Published:** 2025-11-07

**Authors:** Sergio Alexandre Gehrke, Jaime Aramburú Júnior, Tiago Luis Eilers Treichel, Germán Odella Colla, Gustavo Coura, Bruno Freitas Mello, Márcio de Carvalho Formiga, Fátima de Campos Buzzi, Sergio Rexhep Tari, Antonio Scarano

**Affiliations:** 1 Department of Implantology, Bioface/Postgrados en Odontología/Universidad Catolica de Murcia, Montevideo 11100, Uruguay; 2 Department of Biotechnology, Universidad Católica de Murcia, 30107 Murcia, Spain; 3 Department of Physiology and Pharmacology, Pro-Rectorate of Graduate Studies and Research (PRPGP), Universidade Federal de Santa Maria, Santa Maria 97105-900, Brazil; 4 Department of Surgery, Faculty of Medicine Veterinary, University of Rio Verde, Rio Verde 75901-970, Brazil; 5 Department of Implantology, Be Dental School, Universidade do Vale do Itajaí (UNIVALI), São José 88102-700, Brazilmarcio.formiga@bedentalschool.com.br (M.d.C.F.); 6 Department of Pharmaceutical Science, School of Health Sciences, Universidade do Vale do Itajaí (UNIVALI), Itajaí 88302-901, Brazil; 7 Department of Innovative Technologies in Medicine & Dentistry, University of Chieti-Pescara, 66013 Chieti, Italy; sergiotari@unich.it (S.R.T.);

**Keywords:** animal study, bone density, dental implants, histomorphometry, non-invasive imaging, osseointegration, radiographic analysis, RGB pseudocolor, sheep model

## Abstract

Objective: This study aimed to evaluate peri-implant tissue changes during early osseointegration using a combined approach of digital radiographic analysis, RGB pseudocolorization, and histomorphometry in a sheep tibia model. Materials and Methods: Thirty titanium implants were placed in the tibiae of six adult sheep and evaluated at 14 and 28 days post-implantation. Digital periapical radiographs were acquired, grayscale values and RGB channel intensities were measured using Fiji/ImageJ, and compared with histological parameters (bone tissue, collagen, and medullary spaces) quantified from picrosirius–hematoxylin-stained sections. Manual overlay of radiographic and histological images was performed to ensure spatial correspondence of regions of interest. Statistical analyses assessed differences over time and correlations between image data and histological composition. Results: Radiographic grayscale values and histologically measured bone and collagen increased significantly from 14 to 28 days (*p* < 0.01), while medullary spaces decreased (*p* < 0.001), indicating progressive bone formation and matrix maturation. RGB analysis revealed significant increases in green channel intensity and decreases in red channel intensity (*p* < 0.05), while the blue channel remained stable. At 14 days, strong correlations were observed between blue channel intensity and bone tissue (r = 0.81; *p* = 0.015), and between green channel intensity and collagen (r = 0.98; *p* < 0.001). Visual overlays demonstrated alignment between radiographic high-density zones and histologically dense bone regions. Conclusions: RGB pseudocolorized radiographic analysis, correlated with histological findings, offers a non-invasive and reproducible method for early detection of peri-implant tissue maturation. This feasibility correlation study provides a foundation for future investigations integrating imaging, histology, and biomechanical testing.

## 1. Introduction

Osseointegration was defined as the direct contact between bone and the implant surface without the interposition of fibrous connective tissue, is a critical factor determining the long-term success of dental and orthopedic implants [1]. Establishing a stable bone–implant interface ensures mechanical fixation and effective load transfer, both of which are essential for the longevity of osseointegration and optimal clinical outcomes.

Traditionally, osseointegration has been evaluated using histological methods, particularly through the quantification of bone-to-implant contact (BIC%) and bone area fraction occupancy (BAFO%) in non-decalcified sections. These parameters provide detailed information about the extent of bone formation and the quality of the bone–implant interface [2,3,4]. However, histological analysis is inherently destructive, requiring sectioning and processing that preclude further use of samples in biomechanical tests such as removal torque measurements—valuable for assessing implant stability and the strength of newly formed bone tissue surrounding the implant [4,5].

To address these limitations, non-invasive imaging modalities such as digital periapical radiography, cone-beam computed tomography (CBCT), and micro-computed tomography (micro-CT) have been increasingly investigated as alternative methods for estimating peri-implant bone formation and density [6,7,8]. These imaging techniques quantify bone mineral density indirectly through grayscale intensity values or Hounsfield units and have shown significant correlations with both histological and biomechanical parameters in various animal models [9,10].

Despite these promising results, variability in imaging protocols, equipment calibration, and exposure parameters continues to hinder the standardization and comparability of findings across studies [11]. Furthermore, most previous research has focused on conventional histomorphometric parameters such as BIC% and BAFO%, which emphasize the extent of direct bone contact with the implant surface but fail to fully characterize the composition of peri-implant tissues.

The present study takes a different approach by quantitatively assessing the relative proportions of bone tissue, collagen-rich connective tissue, and marrow spaces in histological sections surrounding titanium implants placed in sheep tibiae. Collagen content, indicative of fibrous tissue presence, along with marrow spaces, offers complementary insights into the healing and remodeling processes at the implant site.

By combining histological quantification of tissue composition with digital periapical radiographic imaging—including grayscale and RGB pseudocolor analysis—this study aims to validate a non-destructive, accessible method for estimating peri-implant tissue status. This approach enables the preservation of samples for subsequent biomechanical testing, such as removal torque analysis, thereby maximizing the utility of animal models and aligning with the 3Rs principles (Replacement, Reduction, and Refinement) in ethical animal research [12,13].

Within this context, the study evaluates correlations between radiographic image parameters and histologically measured proportions of bone, collagen, and marrow spaces after 14 and 28 days of healing. The findings aim to contribute to the development of more comprehensive, non-invasive methods for monitoring osseointegration and peri-implant tissue remodeling in preclinical models.

## 2. Materials and Methods

### 2.1. Animals and General Care

Six adult Santa Inês sheep, weighing between 40 and 50 kg and with a mean age of 2 ± 0.5 years, were used in this study. The animals were obtained from the breeding facility of the University of Rio Verde (Rio Verde, Brazil), and all were healthy, previously vaccinated, and clinically evaluated (including hoof and parasite checks) to ensure appropriate physical condition. The sheep were housed in individual pens with free access to water and mineral salt throughout the experiment. Feeding, pre- and postoperative fasting, and animal care were supervised by a veterinarian from the University of Rio Verde School of Veterinary Medicine. All procedures were approved by the Institutional Animal Care and Use Committee (CEUA/UnRV #18/2018).

### 2.2. General Procedures and Anesthesia

Food and water were withheld for 24 h prior to surgery. All surgical procedures were performed under general anesthesia, with continuous veterinary monitoring to minimize pain and stress. Premedication consisted of intramuscular Midazolam 0.3 mg/kg (Hipolabor Farmacêutica, Sabará, Brazil) and Tramadol 2 mg/kg (Hipolabor Farmacêutica, Sabará, Brazil), followed by intravenous Ringer’s lactate solution 5 mL/kg/h (Magazine Médica, Concórdia, Brazil). Anesthesia was induced with Propofol 4 mg/kg IV (Cristália Produtos Químicos Farmacêuticos Ltd.a, São Paulo, Brazil) and maintained with 1% isoflurane after orotracheal intubation. Epidural anesthesia was administered with Lidocaine 4 mg/kg (DFL, Rio de Janeiro, Brazil) and Morphine 0.1 mg/kg (Dimorf—Cristália Produtos Químicos Farmacêuticos Ltd.a, São Paulo, Brazil). Surgical sites were prepared under sterile conditions by shaving, washing, and disinfecting with 10% povidone-iodine.

### 2.3. Implant Placement Surgery

**Figure 1:**
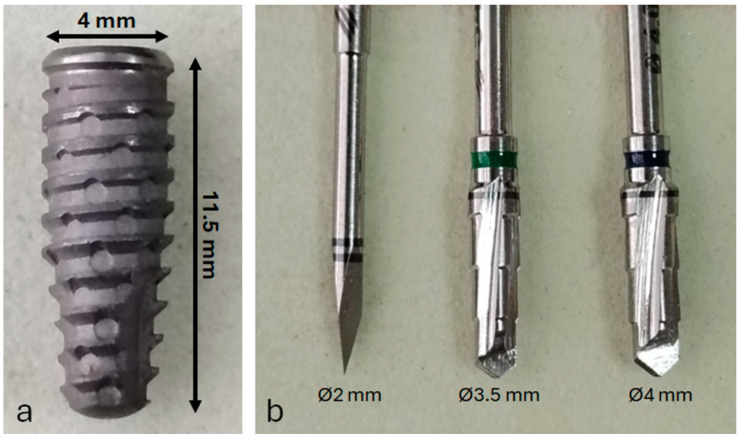
(**a**) Representative image of the titanium dental implant used in this study; (**b**) surgical drill sequence employed for osteotomy preparation prior to implant placement.

A total of 30 commercially pure titanium implants (Grade IV) were placed. All implants had an identical design and surface treatment: conical implants with Morse taper connection (Maestro implant, Implacil/Osstem, São Paulo, Brazil), with 4 mm diameter and 11 mm length (Figure 1a). After soft tissue incision and reflection, the tibial bone was exposed. Osteotomies were prepared using a sequential drilling protocol recommended by the manufacturer (Figure 1b), under continuous irrigation with 0.9% saline solution.

**Figure 2:**
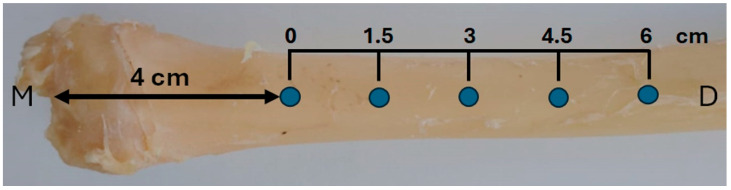
Schematic illustration of implant positioning in the right tibia. Each animal received five implants spaced 1.5 cm apart, starting 4 cm distal to the medial tibial plateau (M). The total implant distribution covered a 6 cm segment of the diaphysis (D).

The implants were installed in the right tibia of each animal, with five implants per tibia, spaced 1.5 cm apart, and the first implant placed 4 cm distal to the joint (Figure 2). Implant insertion was performed manually using a torque wrench, with an insertion torque of approximately 25 ± 2 Ncm. After placement, soft tissues were repositioned and sutured using 4-0 nylon in a simple interrupted pattern.

### 2.4. Postoperative Care, Euthanasia, and Group Allocation

Postoperative analgesia included intravenous Tramadol (2 mg/kg) and oral Meloxicam (0.5 mg/kg) for the first three days after surgery. Antibiotic prophylaxis was provided with intramuscular Oxytetracycline 0.1 mg/kg (Alivira Saúde Animal, Campinas, Brazil) for one week. Daily topical silver sprays were applied to the surgical site to prevent local infection. Water and food were offered immediately after surgery.

Animals were euthanized at two different time points, forming two experimental groups: Group 1, euthanasia 14 days post-implantation; Group 2, euthanasia 28 days post-implantation. Euthanasia was performed using an anesthetic overdose. Immediately afterward, the tibiae containing the implants were harvested and fixed in 10% buffered formalin.

### 2.5. Radiographic Image Acquisition

The harvested tibiae were sectioned into 10 mm thick bone blocks, with the implant centered within the block. Digital periapical radiographs of each sample were acquired before histological processing. A portable digital radiography system (IriX-ray DX 3000; Dexcowin, Seoul, Republic of Korea) was positioned 15 cm from the sample, and images were captured using an RVG First intraoral digital sensor (Trophy, Toulouse, France), with the bone block placed directly on the sensor. Radiographic images were acquired using the Trophy Imaging software (version 7.0.19.3.d1), and colorized versions were generated using the thermal color filter available within the software (color image function).

### 2.6. Histological Processing and Image Acquisition

Bone blocks containing the implants were processed following standard protocols for undecalcified sections. Samples were dehydrated in ascending alcohol concentrations (70% to 100%), embedded in glycol methacrylate resin (Technovit 7200 VLC; Kulzer, Germany), and sectioned transversely with a diamond saw to approximately 120 µm, then ground down to a final thickness of ~30 µm. A central longitudinal section of each implant was stained with picrosirius–hematoxylin. This stain was selected for its ability to clearly distinguish bone from collagenous tissue, as previously reported [4]. Images were acquired using an optical microscope with a high-resolution digital camera (Nikon, Tokyo, Japan) and digital capture system (Image Pro Plus 4.5; Media Cybernetics, National Institute of Health, Bethesda, MD, USA).

### 2.7. Definition of Evaluation Areas in the Images

**Figure 3:**
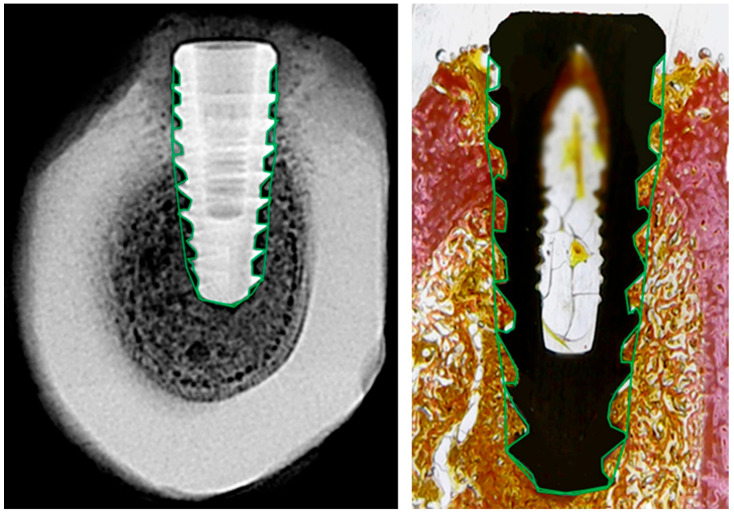
Representative images of radiographic (**left**) and histological (**right**) analysis. The region of interest (ROI) was manually delineated around the full perimeter of the implant, following the bone-implant interface (green outline). This standardized ROI was used in both modalities to extract grayscale density values and histological tissue proportions.

Radiographic and histological analyses were performed by defining a single region of interest (ROI) that encompassed the entire bone-implant interface, including all implant threads, as shown in Figure 3. Although the analysis focused on the interthread regions, where bone remodeling is most pronounced during healing, the ROI encompassed nearly the entire implant, as the threads extend along almost its full length.

The ROI was manually delineated on both sides of each implant to ensure full coverage of the peri-implant area. This standardized approach allowed consistent and reproducible comparisons between radiographic grayscale data and histological tissue composition.

### 2.8. Radiographic Bone Density Evaluation

**Figure 4:**
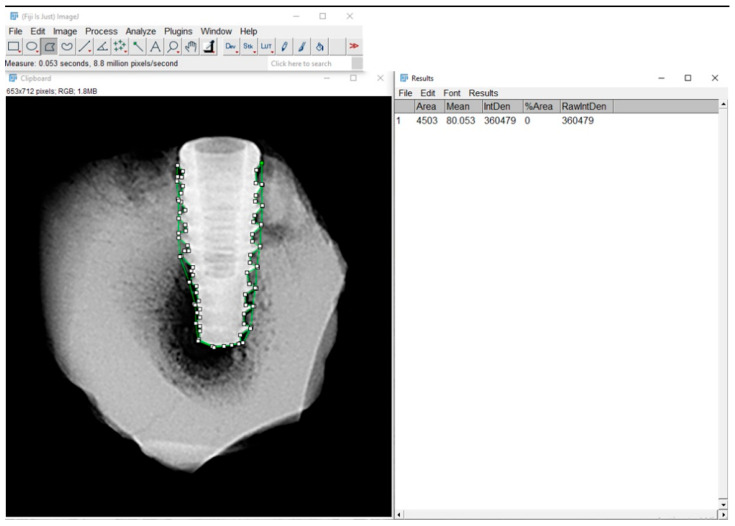
Radiographic analysis of bone density using Fiji/ImageJ software. Region of interest (ROI) was manually delineated around the bone-implant interface. The green-highlighted area in the image corresponds to the ROI.

Grayscale radiographic images were converted to 8-bit and analyzed using Fiji/ImageJ 2.9.0 software (NIH and LOCI, University of Wisconsin, Madison, WI, USA) with a custom macro script. The region of interest (ROI) was manually defined around the entire implant, following the thread contours. Although the analysis emphasized the interthread regions, the ROI effectively covered nearly the entire implant, as the threads extend almost to their full length. The mean gray value was automatically extracted for each ROI and used as an indirect estimate of local bone density (Figure 4).

To assist in data interpretation, gray values were classified according to their typical correspondence with tissue density observed in radiographic images, as presented in Table 1.

This classification enabled an approximate differentiation between tissues with low, medium, and high mineral density, supporting the correlation with histological parameters and aiding in the interpretation of peri-implant bone formation.

### 2.9. Histological Parameters’ Evaluation

**Figure 5:**
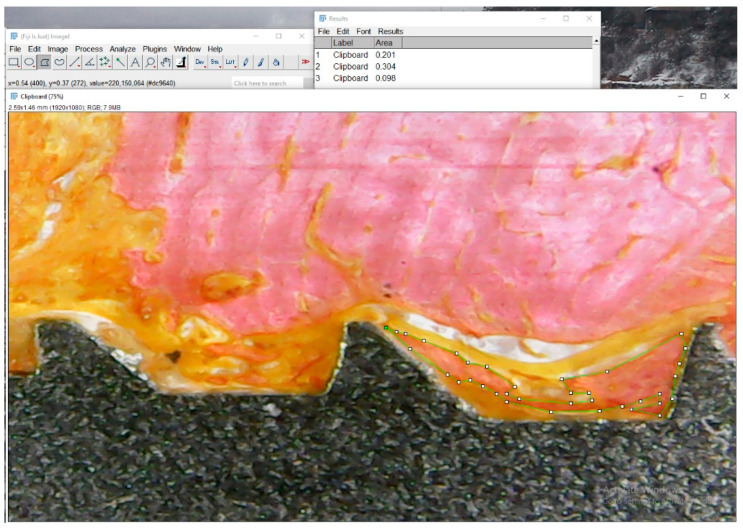
Representative histological image illustrating tissue segmentation using Fiji/ImageJ. In this example, the green-outlined region represents bone tissue (pink area). Collagenous tissue is identified in orange, and the medullary space appears as white. The software was calibrated using the known dimensions of the implant, and the relative areas of each tissue type were measured within predefined regions of interest.

Histomorphometric analysis was performed using Fiji/ImageJ, with the software calibrated based on implant dimensions. The total area around each implant within each thread (Figure 3, right) was defined and considered as 100%. Then, using higher-magnification images, the relative areas occupied by collagen tissue, bone tissue, and marrow space were measured. The results are expressed as percentage values of total area, as illustrated in Figure 5.

### 2.10. Evaluation of Colorized Radiographs in Predetermined Areas

Colorized radiographs with thermal pseudocoloring were used to represent bone density variations in the RGB color space. A LUT (Look-Up Table) was applied, assigning the following color interpretations:

- Red = lower density (less mineralized bone or soft tissue);
- Green = intermediate density;
- Blue = higher density (more mineralized bone).

Quantitative analysis of RGB channels was performed in Fiji/ImageJ. Each image was decomposed into its red, green, and blue channels using the command:

Image > Color > Split Channels, resulting in three separate grayscale images corresponding to each color component.

**Figure 6:**
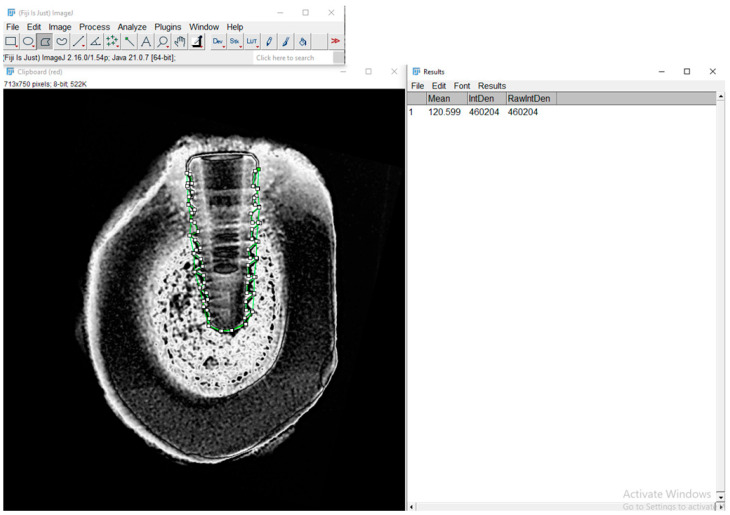
Representative image of the RGB analysis of a pseudocolorized radiograph processed in Fiji/ImageJ. The image shows the red channel used for evaluating the green-marked region of interest (ROI) around the bone-implant interface.

Regions of interest (ROIs) were defined using the Freehand Selections tool, standardized in size, and applied individually to each color channel image (R, G, B). Mean, minimum, and maximum intensity values were extracted using the Analyze > Measure command (Figure 6). The data were exported to spreadsheets for subsequent statistical analysis.

### 2.11. Overlay of Radiographic and Histological Images

Pseudocolorized periapical radiographs were used as base images for spatial alignment with corresponding histological sections. Manual image overlay was performed in Fiji/ImageJ using anatomical landmarks visible in both modalities (e.g., implant contours, cortical boundaries, marrow spaces). This spatial correspondence allowed accurate matching of regions of interest (ROIs) between imaging methods and ensured valid comparisons of radiographic bone density with histological outcomes.

This alignment approach improved precision in ROI selection and enhanced reproducibility in comparative analyses.

### 2.12. Statistical Analysis

Comparisons between the 14- and 28-day groups were performed using unpaired tests, as these represent independent samples from different animals. Parametric (unpaired *t*-test) or non-parametric (Mann–Whitney) tests were applied according to data normality, which was assessed using the Shapiro–Wilk test. No paired analyses were performed. For repeated measures within the same implant (e.g., RGB channel analysis), one-way ANOVA followed by Bonferroni’s multiple comparisons test was applied when data met parametric assumptions. Bartlett’s test was used to assess the homogeneity of variances. Statistical significance was defined as *p* < 0.05. We acknowledge that multiple implants were placed within the same animal, which introduces potential intra-animal correlations (pseudo-replication). While each implant was analyzed separately, future studies should consider mixed-effects modeling with “animal” as a random factor to account for clustered data and improve statistical robustness. All statistical analyses were performed using GraphPad Prism 9 software (San Diego, CA, USA).

## 3. Results

All animals recovered well from surgical procedures without any postoperative complications or adverse events. At the time of sample collection, all implants were clinically stable, with no signs of infection, mobility, or inflammation at the surgical sites.

The bone density, measured by mean grayscale values, showed a statistically significant increase from 14 to 28 days (median 57.5 vs. 96.0; *p* < 0.05, unpaired *t*-test), indicating progressive mineralization over time.

RGB channel analysis revealed significant differences between groups in the red channel intensity, with higher values at 28 days (median 10.0 vs. 7.0; *p* < 0.001, Mann–Whitney test), suggesting changes in bone quality. The green and blue channels showed mixed normality results; appropriate statistical tests were applied accordingly.

Histomorphometric evaluation demonstrated a significant increase in bone tissue percentage at 28 days compared to 14 days (median 38.5% vs. 16.0%; *p* < 0.001, Mann–Whitney test), alongside an increase in collagen percentage (median 10.0% vs. 0.0%; *p* < 0.01). Conversely, medullary space decreased significantly over time (median 25.0% vs. 82.5%; *p* < 0.001).

These findings indicate progressive osseointegration characterized by increased bone formation and mineralization surrounding the implants over the studied periods.

### 3.1. Radiographic Bone Density

Radiographic bone density was assessed by analyzing mean grayscale values (on an 8-bit scale) from standardized radiographic images obtained around dental implants at 14 days (Group 1) and 28 days (Group 2) post-implantation. Higher grayscale values correspond to increased mineralized tissue density.

**Figure 7:**
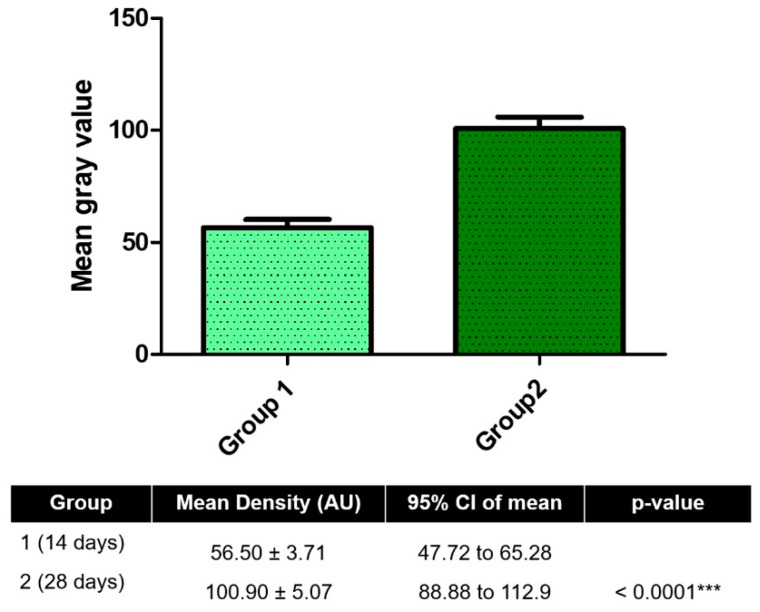
Radiographic analysis of peri-implant bone density at 14 and 28 days post-implantation. Mean gray values (arbitrary units, AU) were measured from standardized radiographs using Fiji/ImageJ software to assess mineralized tissue density around implants. *** *p* < 0.001.

The 28-day group demonstrated a significantly higher mean grayscale value (100.9 ± 5.07) compared to the 14-day group (56.5 ± 3.71) (*p* < 0.0001, unpaired *t*-test), indicating substantial mineralization over time. The mean difference between groups was −44.37 ± 6.28, with a 95% confidence interval of −57.85 to −30.90. No significant difference in variance was observed between the groups (*p* = 0.4289). These results confirm a progressive increase in peri-implant bone density between the two time points, as shown in Figure 7.

### 3.2. RGB Analysis of Pseudocolorized Images

RGB channel intensity analysis of pseudocolorized radiographic images revealed significant differences between groups and among color channels (*p* < 0.0001, one-way ANOVA, F = 43.65, R^2^ = 0.8386). Bonferroni’s post hoc test confirmed that red channel (R) intensities were significantly higher than green (G) and blue (B) at both time points (*p* < 0.001).

Over time, green channel intensity increased significantly from 14 to 28 days (69.79 ± 22.92 vs. 134.00 ± 28.37; *p* = 0.0022), while red channel intensity decreased (139.60 ± 34.42 vs. 105.50 ± 19.64; *p* = 0.0182). Blue channel intensity showed no significant difference between groups (*p* = 0.2884). Bartlett’s test indicated significant variance differences among RGB channels (*p* = 0.0051), suggesting heterogeneity in pixel distribution.

**Figure 8:**
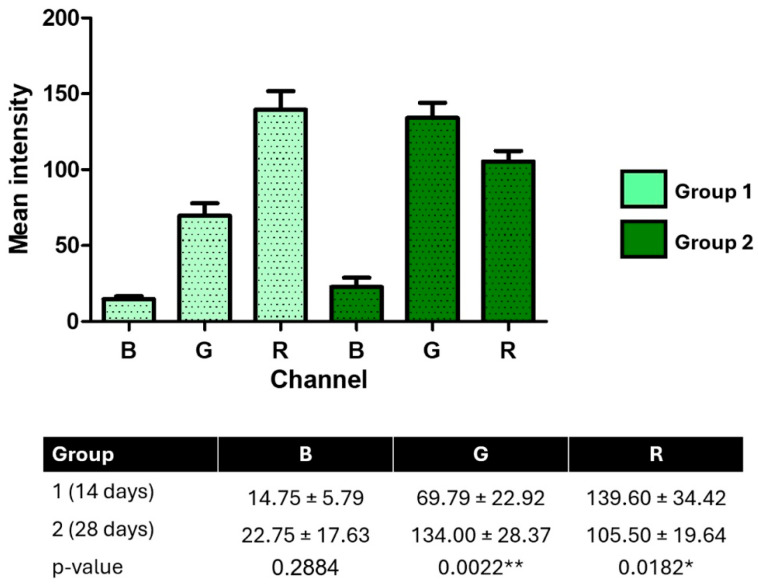
RGB channel intensity analysis of pseudocolorized peri-implant radiographs at 14 and 28 days post-implantation. Mean pixel intensities for blue (B), green (G), and red (R) channels were extracted from standardized images using Fiji/ImageJ. Data are expressed as mean ± SD. Statistical differences were determined using one-way ANOVA with Bonferroni’s post hoc test and unpaired *t*-tests. * *p* < 0.05; ** *p* < 0.01.

These findings reflect dynamic shifts in tissue composition at the bone–implant interface, with changes in color channel intensities potentially corresponding to differences in mineral content and collagen organization over time (Figure 8).

### 3.3. Histomorphometric Analysis

Histomorphometric analysis of peri-implant tissues revealed significant temporal changes between 14 and 28 days post-implantation. The percentage of bone tissue (BT) increased significantly from 3.63 ± 3.25% at 14 days to 17.38 ± 8.50% at 28 days (*p* = 0.0057), indicating new bone formation. Likewise, collagen tissue (CT) showed a marked increase, rising from 15.75 ± 5.04% to 40.38 ± 14.55% (*p* = 0.0030), suggesting active extracellular matrix production and tissue maturation.

**Figure 9:**
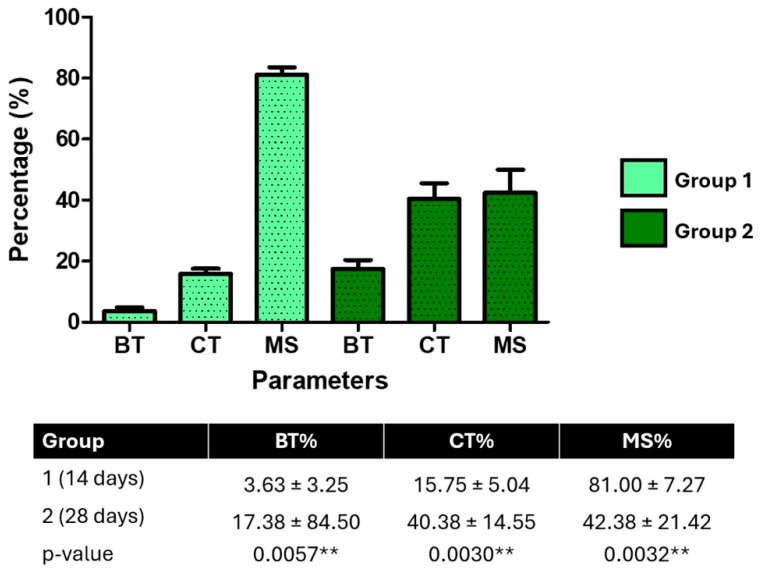
Histomorphometric quantification of peri-implant tissue composition at 14 days (Group 1) and 28 days (Group 2). The percentage of bone tissue (BT), collagen tissue (CT), and medullary spaces (MS) was measured from histological images using Fiji/ImageJ. BT and CT increased significantly over time, while MS decreased (*p* < 0.01, unpaired *t*-tests), indicating progressive bone maturation. ** *p* < 0.01.

In contrast, the medullary space (MS) significantly decreased over time, from 81.00 ± 7.27% to 42.38 ± 21.42% (*p* = 0.0032), reflecting progressive bone infill and structural consolidation around the implant. These histological findings provide quantitative evidence of ongoing osseointegration and bone remodeling during the study period (Figure 9).

### 3.4. Visual Correlation Between Radiographic and Histological Images

**Figure 10:**
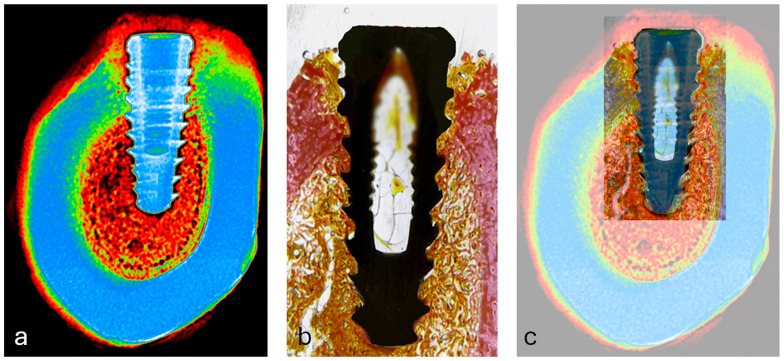
(**a**) Pseudocolorized periapical radiograph used as the reference image for alignment; (**b**) corresponding histological section showing bone-implant interface; (**c**) overlay of radiographic and histological images using anatomical landmarks (implant contour, cortical bone, and marrow spaces) for manual spatial alignment in Fiji/ImageJ.

To qualitatively assess spatial correspondence between radiographic grayscale intensities and histological tissue components, manual overlay of pseudocolorized radiographs and matched histological sections was performed (Figure 10). Visual alignment using anatomical landmarks (implant geometry, cortical boundaries, and marrow spaces) allowed for identification of shared regions of interest (ROIs) across both modalities.

This overlay revealed that regions of higher grayscale intensity (typically represented by warmer colors in the pseudocolor scale) visually coincided with areas of dense bone tissue in histology, while low-intensity zones matched marrow spaces. Although this alignment did not produce quantitative output, it supported the selection accuracy of ROIs and provided visual validation of the image-based mineral density analysis.

### 3.5. Correlation Between RGB Channel Intensities and Tissue Composition

Pearson correlation analyses were performed to assess the relationship between RGB channel intensities of pseudocolorized images and peri-implant tissue composition (bone tissue [BT], collagen tissue [CT], and medullary spaces [MS]) at 14 and 28 days. At 14 days, significant positive correlations were found between blue channel intensity and BT (r = 0.81, *p* = 0.015) and between green channel intensity and CT (r = 0.98, *p* < 0.001). No significant correlation was observed between red channel intensity and MS (r = −0.34, *p* = 0.41). At 28 days, green channel intensity remained significantly correlated with CT (r = 0.82, *p* = 0.013), while correlations between blue channel and BT (r = 0.37, *p* = 0.37) and red channel and MS (r = 0.70, *p* = 0.056) were not statistically significant.

These findings indicate that blue and green channel intensities may effectively reflect bone and collagen presence during early healing stages.

## 4. Discussion

This study evaluated the initial osseointegration process around dental implants through a combined approach of digital radiographic analysis, pseudocolorization by RGB channels, and histomorphometric quantification, in two experimental times (14 and 28 days). The choice of these periods was based on evidence from the literature demonstrating that the first weeks after implant installation are critical for bone deposition and extracellular matrix formation [4,14,15,16]. These intervals were selected to capture distinct and contrasting phases of early osseointegration, allowing for the evaluation of initial bone remodeling and matrix maturation. Future studies could include additional time points, such as 7, 21, or 42 days post-implantation, to provide a more comprehensive understanding of the temporal dynamics of bone healing and osseointegration progression over time. Unlike studies that compare implant surfaces or macrogeometries [17,18,19], our methodological approach sought to correlate histological parameters with metrics extracted from digital images, to explore the potential of image analysis as a complementary and non-invasive tool for monitoring osseointegration. This approach allowed for the integrated observation of structural and compositional changes in peri-implant tissue over time, offering a comprehensive perspective on the dynamics of bone repair. Analysis of the results obtained in this study demonstrated significant and progressive changes in bone density, tissue composition, and peri-implant mineralization between 14 and 28 days after implantation. These findings corroborate the current literature on the dynamic processes of osseointegration and bone remodeling around implants [3,4,15].

A significant increase in bone density values, measured by gray scales on digital radiographs, indicates an increase in the content of mineralized tissue over time [20,21]. This is consistent with previous studies demonstrating that bone mineralization and filling of marrow spaces occur in the first weeks after implant placement, reflecting an efficient biological response to the presence of the implanted material [5,6,22].

RGB analysis of the pseudocolorized images revealed distinct changes in the intensity of the color channels, especially with increasing intensity in the green channel and decreasing intensity in the red channel over time. These changes may reflect the replacement of marrow tissue by mineralized tissue and collagen, since the color patterns in pseudocolorized images are associated with the composition and density of the tissue [23]. The blue channel, on the other hand, showed no significant variations, suggesting less sensitivity to tissue remodeling in this context. This can be explained by the relatively short evaluation time (14 and 28 days), in which initial changes in collagen matrix deposition and replacement of marrow spaces predominate, while denser bone mineralization—which could be better reflected by this channel—tends to occur in more advanced stages of osseointegration. Thus, the results obtained are consistent with the experimental model adopted and reinforce the specificity of each color channel in detecting different tissue components.

Histomorphometric evaluation confirmed these findings, showing a significant increase in the proportion of bone tissue and collagen, accompanied by a marked reduction in marrow spaces. This pattern indicates not only bone matrix deposition, but also tissue organization and maturation, essential steps for long-term implant stability. The increased presence of collagen is also indicative of extracellular matrix formation and support for subsequent mineralization [9,24].

Furthermore, correlation analysis between RGB channel intensities and histomorphometric parameters provided additional insights into the biological interpretation of the imaging data. At 14 days, strong and statistically significant correlations were observed between the blue channel and bone tissue (r = 0.81; *p* = 0.015), as well as between the green channel and collagen (r = 0.98; *p* < 0.0001), suggesting that pseudocolor analysis can reliably reflect tissue organization in the early stages of osseointegration. Although these associations were less consistent at 28 days—possibly due to increased tissue heterogeneity or advancing maturation—the correlation between the green channel and collagen remained significant (r = 0.82; *p* = 0.012), indicating its usefulness as a possible non-invasive marker of extracellular matrix deposition. The red channel, hypothesized as a correlate of medullary spaces, showed weak and non-significant correlations at both times, which suggests limitations in its isolated applicability in this type of analysis.

The choice of intervals of 14 and 28 days was recommended precisely to capture distinct and yet contrasting phases of osseointegration, allowing for the clear identification of tissue changes both in radiographs and in histological analysis. These periods represent critical windows in the bone repair process, in which the transition between marrow tissue, collagenous matrix, and mineralized tissue can still be clearly evidenced [25]. If longer periods were used, it is likely that only bone tissue would predominate around the implants, with a significant reduction in the differences between the tissues present, making comparative analysis and temporal interpretation of the results difficult.

Collectively, the results suggest that, even over relatively short periods (28 days), there is significant progression in the osseointegration process, involving both structural (mineral density) and biological aspects (collagenous matrix formation and bone filling) [26,27]. These findings reinforce the importance of early and quantitative monitoring of bone regeneration around implants, contributing to the development of evidence-based clinical strategies.

In addition to demonstrating the feasibility of radiographic–histologic correlation [28,29], the present workflow suggests that quantitative analysis of digital periapical radiographs could serve as a surrogate tool for assessing early osseointegration parameters. By extracting objective gray-level and RGB-based metrics directly from radiographic images, it may be possible to reduce the number of histological sections required for evaluation and to preserve specimens for subsequent biomechanical (torque removal) testing. This integrated analytical approach allows more information to be obtained from each animal, contributing to more efficient and ethical preclinical protocols in accordance with the Reduction principle of the 3Rs.

Limitations of the present study include the limited number of animals, the short observation period, and the lack of functional or biomechanical assessment of implant stability. Additionally, the clustered nature of the data, with multiple implants placed within the same animal, introduces pseudo-replication, meaning that implants within an individual sheep are not fully independent. Although each implant was analyzed separately, intra-animal correlations may have influenced variability. Future studies should apply mixed-effects modeling (with animal as a random effect) to account for within-subject dependency and improve statistical robustness.

Future studies with longer periods, different biomaterials, functional approaches, and clinical validation are needed to deepen the understanding of the mechanisms involved in osseointegration and validate the clinical application of RGB analysis in digital radiographic images. As this was a preliminary feasibility study primarily designed to explore the potential of radiographic parameters as indicators of early osseointegration, a formal sample size calculation was not performed a priori. Nevertheless, the present results provide valuable effect size estimates that can guide future power analyses and the optimization of experimental designs. Such data-driven planning is consistent with ARRIVE and NC3Rs recommendations, supporting more efficient protocols that achieve statistical robustness while minimizing animal use.

Finally, the manual delineation of regions of interest (ROIs) represents a potential source of variability, despite efforts to standardize the procedure. Future studies could explore the integration of artificial intelligence (AI)-based software to assist in ROI definition, which may enhance reproducibility, reduce observer bias, and streamline the analysis process [30,31,32]. Such approaches could complement the current methodology, allowing more efficient and objective assessment of peri-implant bone changes.

## 5. Conclusions

This study demonstrates the feasibility of integrating digital radiographic analysis, RGB pseudocolorization, and histomorphometry to quantitatively characterize early peri-implant tissue changes. Between 14 and 28 days after implantation, significant increases in mineral density, bone tissue, and collagen were observed, accompanied by a reduction in marrow spaces. The correlations between RGB channels and histological components suggest that pseudocolor analysis can serve as a non-invasive complementary tool to monitor peri-implant tissue maturation during the initial phases of osseointegration.

While biomechanical validation was not performed, this correlation framework establishes a foundation for future studies that aim to combine imaging, histology, and mechanical assessments, thereby supporting the development of quantitative and reproducible approaches for evaluating early bone regeneration around implants.

## Figures and Tables

**Table 1:** Interpretation of grayscale values in radiographic images.

Gray Value Range	Typical Meaning
0–50	Black or very dark (empty spaces or low-density tissue)
50–150	Intermediate tones (trabecular bone or mixed tissue)
150–255	Light or white (cortical bone or high-density tissue)

## Data Availability

The original contributions presented in the study are included in the article; further inquiries can be directed to the corresponding authors.
